# Failure of alemtuzumab therapy in three patients with MOG antibody associated disease

**DOI:** 10.1186/s12883-022-02612-6

**Published:** 2022-03-09

**Authors:** Sinali O. Seneviratne, Mark Marriott, Sudarshini Ramanathan, Wei Yeh, Fabienne Brilot-Turville, Helmut Butzkueven, Mastura Monif

**Affiliations:** 1 Curtin University, Kent Street, Bentley, Perth, WA 6102 Australia; 2 Department of Neurology, Royal Melbourne Hospital, 300 Grattan Street, Parkville VIC 3050, Australia; 3 Translational Neuroimmunology Group, Kids Neuroscience Centre, The Kids Research Institute at the Children’s Hospital, Westmead, NSW Australia; 4 Sydney Medical School, University of Sydney, Sydney, NSW Australia; 5 Department of Neurology, Concord Hospital, Sydney, Australia; 6 Department of Neurology, Alfred Hospital, 55 Commercial Rd, Melbourne, VIC 3004 Australia; 7 Department of Neurology, Eastern Health, Box Hill, Victoria, Australia; 8 Department of Neuroscience, Monash University, Clayton, VIC Australia

**Keywords:** Multiple sclerosis, Demyelination, Autoimmune, MOG antibody, Encephalomyelitis

## Abstract

**Background:**

Myelin Oligodendrocyte Glycoprotein antibody-associated disease (MOGAD) is most classically associated in both children and adults with phenotypes including bilateral and recurrent optic neuritis (ON) and transverse myelitis (TM), with the absence of brain lesions characteristic of multiple sclerosis (MS). ADEM phenotype is the most common presentation of MOGAD in children. However, the presence of clinical phenotypes including unilateral ON and short TM in some patients with MOGAD may lead to their misdiagnosis as MS. Thus, clinically and radiologically, MOGAD can mimic MS and clinical vigilance is required for accurate diagnostic workup.

**Case presentation:**

We present three cases initially diagnosed as MS and then treated with alemtuzumab. Unexpectedly, all three patients did quite poorly on this medication, with a decline in their clinical status with worsening of expanded disability status scale (EDSS) and an increasing lesion load on magnetic resonance imaging of the brain. Subsequently, all three cases were found to have anti-MOG antibody in their serum.

**Conclusions:**

These cases highlight that if a patient suspected to have MS does not respond to conventional treatments such as alemtuzumab, a search for alternative diagnoses such as MOG antibody disease may be warranted.

## Background

Multiple sclerosis (MS) is a demyelinating disease of the central nervous system with a characteristic clinical and radiological profile [1]. It can be misdiagnosed with other demyelinating disorders due to overlapping imaging features and clinical presentations, which can vary widely between different patients and within the same patient over time [2].

One of the immunomodulatory medications for the treatment of MS is alemtuzumab, a monoclonal antibody that leads to reduced levels of T and B lymphocytes via its targeting of the cell-surface glycoprotein CD52 [3]. In the CAMMS223 study [4], patients with relapsing–remitting MS (RRMS) and Expanded Disability Status Scale (EDSS) score of 0–3 were randomized to treatment with alemtuzumab or interferon beta 1a (IFNB-1a). At 3 years, the annualised relapse rate was significantly reduced by 70% in patients who received alemtuzumab compared to IFNB-1a [4]. Similarly confirmed disability worsening (CDW) was reduced by 70% in patients receiving alemtuzumab compared with those in the IFNB-1a group [4], highlighting the very effective therapeutic potential of alemtuzumab in the treatment of RRMS. A well-established adverse effect of alemtuzumab therapy is the emergence of secondary autoimmune conditions such as Graves’ disease and immune thrombocytopenia [3]. Moreover, it has recently been reported that alemtuzumab can also induce diffuse alveolar bleeding [5].

MOG antibody-associated disease (MOGAD) is one of the demyelinating diseases that can be misdiagnosed as MS [6]. Typically, these patients present with optic neuritis (ON) (bilateral ON more frequent than unilateral ON), acute demyelinating encephalomyelitis (ADEM), transverse myelitis (TM), and cortical seizures in the absence of brain lesions consistent with MS [7]. While bilateral ON and longitudinally extensive TM are more characteristic presentations in MOGAD, the phenotypes that do overlap with MS include unilateral ON and short TM [8]. MOGAD is an autoimmune condition characterised by the synthesis of IgG antibodies against MOG, a glycoprotein found on the outer membrane of the myelin sheath in the central nervous system [9].

We present three cases of anti-MOG encephalomyelitis initially diagnosed as MS and treated with alemtuzumab followed by multiple relapses and worsening disability on alemtuzumab. The diagnosis of MOGAD was finally established with antibody testing by flow cytometry live cell-based assay. These cases highlight that (a) MOGAD can be misdiagnosed as MS and, (b) alemtuzumab may be ineffective in MOGAD.

## Case presentation

### Case 1

A Caucasian woman in her early thirties presented with numbness of left arm and tongue as well as left-sided ptosis followed by another episode of left arm, leg, and trunk numbness, urinary frequency, and vertigo five months later. Her past medical history included alopecia totalis, vitiligo, asthma, and psoriasis. She was diagnosed with MS and treated with beta interferon followed by natalizumab. Due to poor response, she was subsequently treated with two cycles of alemtuzumab 12 months apart.

Unexpectedly, the patient experienced several clinical relapses following treatment with alemtuzumab. Fourteen months after the second cycle of alemtuzumab, she experienced dizziness and nausea. The neurological examination revealed a convergent eye spasm. She was treated with intravenous steroids, with a good response. Five months after this initial relapse, she experienced another relapse, though this second one was of a milder nature, and involved a flare-up of her “dizzy” (vertiginous) symptoms and some nausea. Physical examination following the second relapse demonstrated convergent eye spasm, and a positive Romberg’s test, and reduced reflexes. Her visual acuity was 6/5 bilaterally. The muscle power was 4 + to 5 in all four limbs. Like the first, her second relapse was also treated with 5 days of 1 g/day intravenous methylprednisolone.

A few weeks later, she experienced further new symptoms which were auditory in nature characterised by sporadic noises in the left ear which had a “banging” and “musical” quality, as well as short and repetitive pieces of music playing in her head. She reported that the sounds were louder at night and prevented her from sleeping. She claimed that the sounds also affected her hearing, as they drowned out external noise. Furthermore, the patient mentioned having left-sided temporal and occipital headaches which had been persisting for two months. She had also been experiencing reduced sensation to the right lower limb for the previous three weeks, however, there was no motor deficit. She was treated with diazepam, although it provided little relief symptomatically. She also visited an ear nose and throat specialist who confirmed that there was no local ear problem. In response to these new symptoms, she was commenced on ocrelizumab and 1000 mg of methylprednisolone was administered intravenously for a period of three days followed by a gradual tapering dose of oral prednisolone. Two weeks after completing her first dose of ocrelizumab, she presented with a relapse characterised by left eye blepharospasm, left ON, and vision in the left eye which had reduced to 6/60. The degree of disc swelling was not documented during this encounter. Convergent eye spasm was still present, as well as partial sixth nerve palsy. Additionally, she demonstrated ataxia and absent reflexes in both the arms and legs. Her EDSS deteriorated from two to five. Further investigations including MRI and anti-MOG antibody testing were carried out due to her deteriorating condition.

**Fig. 1:**
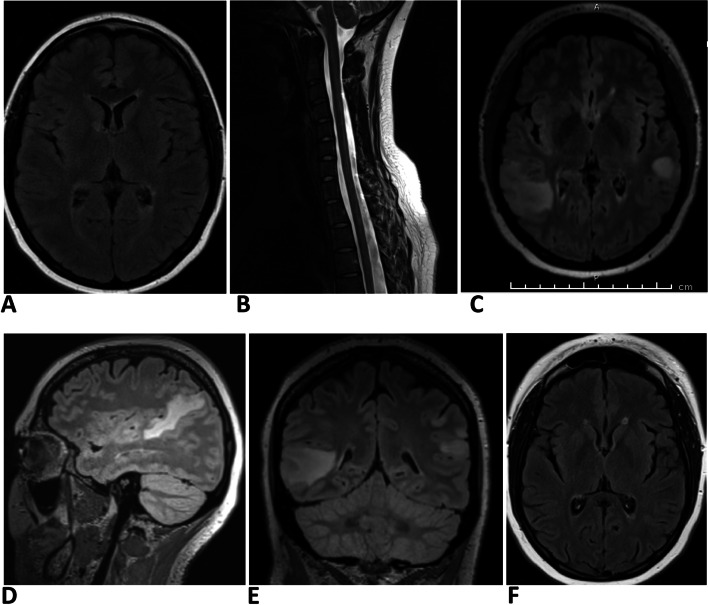
Serial MRI changes of case 1. **A** & **B **MRI brain scan (axial FLAIR sequence) and cervical spine (sagittal T2 sequence) at the time of the diagnosis of MS. Note few periventricular brain lesions and ill-defined focal areas of T2 hyperintensity at C4, C5, and C6 levels. **C**, **D**, **E **MRI brain scan at the third relapse following Alemtuzumab therapy. Axial, sagittal, and coronal FLAIR sequences are shown here. Note bilateral large supratentorial lesions with ill-defined borders, mostly juxtacortical in location, with accompanying oedema. **F **Axial FLAIR image of the brain following mycophenolate therapy. Note the resolution of large juxtacortical lesions seen in **C**,**D**,**E** leaving a few periventricular residual lesions

At the first presentation, the MRI of the brain demonstrated multifocal supra-tentorial T2 hyperintensities and one infra-tentorial hyperintensity, whilst her spinal imaging showed multiple lesions in the cervical and thoracic spinal cord (Fig. 1A, B). Her cerebrospinal fluid was not tested by the treating neurologist at that stage.

Twenty months after the second cycle of alemtuzumab, further neuroimaging with MRI demonstrated multiple, bilateral, supra-tentorial lesions distributed mostly in the juxtacortical locations with overlying cortex involvement and oedema accompanied by mass effect, highly atypical for MS (Fig. 1-C, D, E). There was no contrast enhancement or diffusion restriction. Increased T2 signal and enhancement of the left optic nerve consistent with ON were also noted.

Due to the radiological brain abnormalities and optic neuritis at the last relapse, the possibility of MOGAD was considered. The patient experienced a relapse two weeks after her first dose of ocrelizumab, and two days after the relapse her MOG antibody test was sent off. This decision to get her tested for MOGAD was made before her treatment with IVIG commenced. Her serum anti MOG antibody returned positive by a live cell-based assay. Her serum aquaporin 4 antibody was negative. As a result of this new diagnosis, she was continued on immunosuppression therapy with ocrelizumab as well as mycophenolate, and following this, a new MRI brain showed significantly improved lesion load (Fig. 1F).

The patient has ongoing follow-up for her MOGAD. Following this severe relapse after alemtuzumab, she was treated with five days of intravenous steroids, as well as five days of intravenous immunoglobulin (IVIG) with dramatic improvement clinically and on MRI scanning. Her vision improved to visual acuity 6/6 bilaterally, with this excellent visual recovery consistent with what is seen in MOGAD. However, reflexes remained absent in both the arms and legs and some ataxia was still present. Overall, her condition was much improved. In addition to the six-monthly ocrelizumab infusions, she was also placed on mycophenolate and high dose oral steroids. Her treatment course was complicated by severe left V1 and V2 herpes zoster which was treated with intravenous aciclovir. This settled and she continues on maintenance ocrelizumab and mycophenolate. The steroids were weaned slowly over 6 months. She remains in remission 24 months after the last relapse.

### Case 2

A Caucasian woman in her mid-twenties was diagnosed with MS following an episode of bilateral ON followed by recurrent right-sided ON. She had no significant past medical history. Following this diagnosis, she was treated with beta interferon for two months, but treatment was ceased as the patient was experiencing side effects. Five months later she experienced a relapse characterised by left leg weakness, and a further two months after the relapse, an MRI spine demonstrated new demyelinating lesions, thus she was put back on beta interferon.

During the following month, the patient had another relapse characterised by left leg weakness, and this was treated with three days of intravenous methylprednisolone. Six months onwards, beta interferon was once again stopped due to its side effects. However, within two months she had a further relapse which was once again characterised by left leg weakness, this was treated with intravenous steroids, and in addition, she was once again placed on beta interferon. Over the course of the following 10 months, she experienced three more relapses which included two episodes of left leg weakness, and one episode of right eye ON. Four months later she was commenced on fingolimod therapy, however, this was discontinued due to side effects such as bradyarrhythmia and shortness of breath. Over the next three years, the patient was not treated with any immunomodulatory medications. During this time period, she experienced seven relapses which were managed with intravenous methylprednisolone.

Subsequently, she began treatment with glatiramer acetate, and this was stopped as the patient reported having several falls whilst on it and feeling generally unwell while on glatiramer acetate. After months of not being on any disease-modifying therapy, she was commenced on her first round of alemtuzumab. Unexpectedly, within the week of receiving alemtuzumab, she experienced another relapse and her EDSS score deteriorated from two to six with worsening left sided weakness and lost her ability to walk. Physical examination post alemtuzumab treatment revealed a positive Romberg’s test, right visual acuity of 6/9 with pinhole correction, left visual acuity of 6/12 with pinhole correction, slow horizontal saccades, upper limb power of 5/5 bilaterally, and reduced lower limb power of 3/5 on the left side and 4/5 on the right side in a pyramidal distribution.

She did not receive the second course of alemtuzumab. Ten months after the initial course of alemtuzumab, she experienced another relapse, thus she was switched over to treatment with natalizumab and fampridine. In particular, she was experiencing spasms of the left shoulder muscles, and although this had been going on for the last six years, it had recently begun to affect her quality of life. In response to these symptoms, she was injected with 100 units of botulinum toxin into her left upper trapezius, rhomboids, and infraspinatus muscles. She was also noted to have an unsteady gait. However, at this stage, with natalizumab and fampridine, her condition improved, as 6 months after treatment commenced, she demonstrated a visual acuity of 6/5 bilaterally with correction and lower limb muscle power of 4 + /5 on the left side, and 5-/5 on the right side.

Ten months into her treatment with natalizumab, the patient displayed ongoing worsening of her condition characterised by an unsteady gait, poor bladder control, bilateral lower limb weakness which was worse on the left side, and lower limb spasticity.

**Fig. 2:**
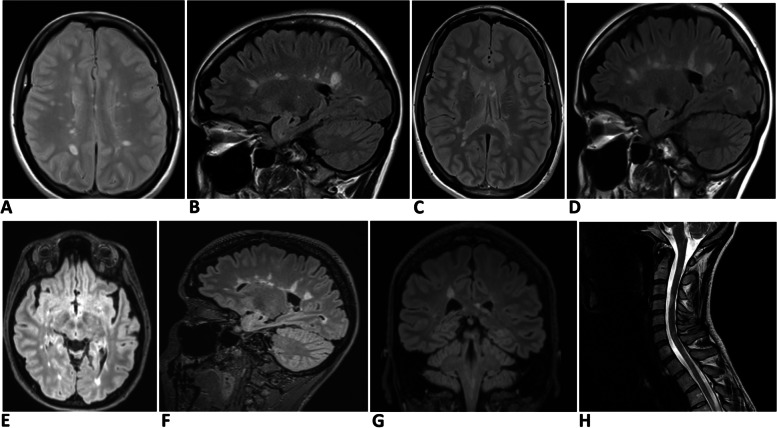
Serial MRI changes of case 2. **A** & **B **Brain scan (axial T2 and sagittal FLAIR sequences respectively) at the time of the diagnosis of MS. Note multiple pericallosal lesions consistent with Dawson’s fingers. **C** & **D **Brain scan (axial T2 and sagittal FLAIR sequences respectively) six years later when Alemtuzumab therapy was commenced. Note changes similar to **A** & **B**. **E**–**G **Images when MOG antibody test was found to be positive. **E** (brain axial FLAIR), **F** (sagittal FLAIR), and **F** (coronal FLAIR) shows multiple pericallosal lesions. **H** (sagittal T2 spine) shows multiple lesions at cervico-medullary junction, C2, C4, C6, and upper thoracic level

MRI brain scan at the first presentation revealed multiple supratentorial lesions (Fig. 2A, B). When her condition deteriorated following alemtuzumab therapy, the MRI scan was repeated. Figure 2C and D show brain MRI changes at that stage.

Twenty-one months after the first dose of alemtuzumab, the patient’s MOG antibody test returned positive, and she was diagnosed with MOGAD. Figure 2E, F, and G show MRI abnormalities at this stage. Her serum aquaporin 4 antibody was negative.

Upon diagnosis of MOGAD, natalizumab and fampridine therapy was ceased and she began treatment with rituximab instead. Following her first rituximab infusion, the patient noticed a decline in her balance as she had a few falls, and she also felt increasingly fatigued. Consequently, she underwent plasma exchange, with an oral course of prednisolone (30 mg weaning over 2 months) with significant improvement in her condition and ability to be able to stand and take 100 steps, something that she had not been able to do for at least 12 months. At the last follow up 7 months after establishing the diagnosis of MOGAD, she reported gait deterioration and frequent falls. Examination revealed bilateral lower limb weakness and hyperreflexia.

### Case 3

A Caucasian woman in her late thirties presented with optic neuritis in 1998 and subsequently a diagnosis of RRMS was established in 1999. The patient entered our service in 2013 after she had ceased natalizumab due to JC virus antibody positivity. The EDSS score was 3.0 and she was switched to fingolimod but reported feeling unwell with MRI disease activity but no EDSS change. She was switched to alemtuzumab and had her first full course in May 2015. In August 2015 she experienced a severe relapse with resultant EDSS score change to 6.5. She recommenced natalizumab in 2016 and remained on this medication for two years with gradual improvement back to EDSS score of 4.0 by May 2017. Her MRI brain was abnormal, and her MRI cord showed multiple small lesions. This is concordant with the recently described MOGAD pattern [10, 11]. In July 2016 she tested positive for MOG antibody.

She was changed to ocrelizumab in late 2017 and relapsed severely in early 2018 with EDSS score increased to 6.0 and a further spinal cord relapse with an EDSS increase to 7.0 in August 2019. At this time, she had a visual acuity of 6/24 in both eyes with no disturbance of eye movements. She had a mild action tremor in both arms, mild pyramidal-type left-hand weakness, left and right pyramidal-type leg weakness with 3/5 hip flexion strength bilaterally, severe weakness in left knee flexion 1/5, and right knee flexion 4/5. She was then recommenced on natalizumab, mycophenolate and high dose oral prednisolone, which was gradually weaned.

**Fig. 3:**
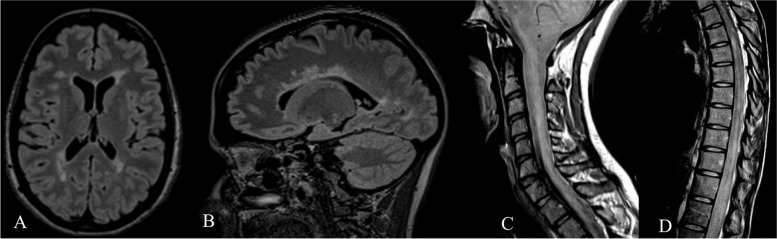
MRI of case 3 following alemtuzumab and subsequent relapse. **A** & **B **Brain imaging (axial and sagittal FLAIR sequences, respectively) with periventricular and subcortical lesions. **C **Cervical cord imaging (sagittal proton density-weighted sequence) with diffuse longitudinally extensive hyperintense signal throughout the cervical cord. D Thoracic cord imaging (sagittal proton density-weighted sequence) with multifocal cord lesions

At the initial presentation, her MRI demonstrated brain lesions and later cord lesions were noted. Her cerebrospinal fluid result was not available. Initially, there were short segment spinal lesions on the MRI. When she experienced a severe relapse three months after alemtuzumab therapy, her MRI demonstrated a very long cervical cord lesion, five thoracic cord lesions, and a moderate brain lesion burden (Fig. 3A, B, C, D). Approximately nine months later, a serum sample showed a positive MOG antibody test. Her serum aquaporin 4 antibody was negative. Two months later, a repeat MRI brain revealed 2 new lesions, further evidence of the failure of alemtuzumab.

The diagnosis of MOGAD was established 14 months following alemtuzumab therapy. Since then, she has been followed up for 4 years and treated with natalizumab followed by ocrelizumab and more recently mycophenolate and oral prednisolone. At the last follow-up, she was moderately disabled with an EDSS score of 6.0.

## Discussion and conclusions

**Fig. 4:**
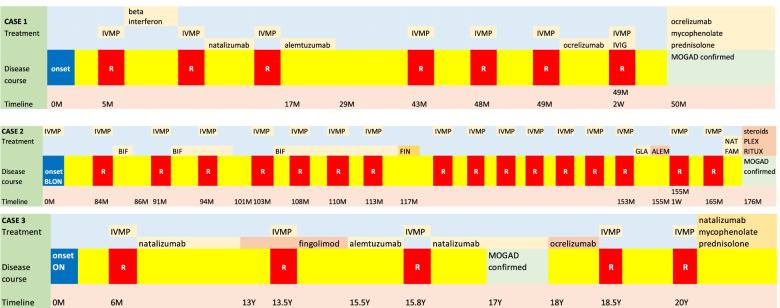
Disease trajectory of the patients. This figure illustrates disease onset and relapses up to the diagnosis of myelin oligodendrocyte glycoprotein antibody associated disease and beyond in relation to the timeline. Changes in the treatment are also shown along the trajectory. ALEM = alemtuzumab; BIF = beta interferon; BLON = bilateral optic neuritis; CIS = clinically isolated syndrome; FAM = fampridine; FIN = fingolimod; GLA = glatiramer acetate; IVIG = intravenous immunoglobulin; IVMP = intravenous methyl prednisolone; M = months; MOGAD = myelin oligodendrocyte glycoprotein antibody-associated disease; NAT = natalizumab; ON = optic neuritis; PLEX = plasma exchange; R = relapse; RITUX = rituximab; W = weeks; Y = years

We report three cases of MOGAD with similar characteristics as summarised in Table 1. Figure 4 characterises the clinical trajectory of all three patients. All are young females initially diagnosed with MS and later treated with alemtuzumab. Two patients presented with ON and demyelinating lesions on MRI brain, whilst the other presented with TM. Multiple relapses were experienced by all three patients while on treatment for MS, including alemtuzumab. MOG antibody returned positive after a latent period following alemtuzumab therapy in the first case. Following the confirmation of MOGAD, all patients were treated with ongoing immunosuppression, with improvement in clinical status. Additionally, one patient was also treated with plasma exchange for which she responded well.

**Table 1:** Summary of clinical characteristics, investigations, and ‘red flags’ of diagnosis in all three patients

	Case 1	Case 2	Case 3	Red flags
**Clinical characteristics**				
Presenting phenotype	Sensory-left arm, tongue	Bilateral optic neuritis	Optic neuritis	Initial presentation with bilateral optic neuritis and relapse with unilateral optic neuritis is highly suggestive of MOGAD Relapse with optic neuritis and transverse myelitis also favours MOGAD rather than MS ADEM phenotype is more common in childhood MOGAD
Relapse phenotype	Unilateral optic neuritis, myelitis, brainstem involvement	Unilateral optic neuritis, transverse myelitis	Bilateral optic neuritis, transverse myelitis	
Disease course	Relapsing	Relapsing	Relapsing	
Optic neuritis	Unilateral (relapse)	Bilateral (onset), unilateral (relapse)	Bilateral	
ADEM phenotype	Yes- on relapse	No	No	
Myelitis	Yes	Yes	Yes	
Brainstem involvement	Yes	No	No	
Cerebellar involvement	No	No	No	
Response to steroids	Recovery from relapse	Recovery from relapse	Recovery from relapse	
Associated autoimmune diseases	Yes	No	No	
**MRI brain**				
Supratentorial lesions at onset	Yes	Yes	Yes	Dawson’s fingers, U-fibre lesions, and periventricular lesions favour MS ADEM-type lesions favour MOGAD. Optic nerve involvement is pre-chiasmatic and longitudinally extensive in MOGAD. Perineural and periorbital enhancement favours MOGAD. 15% of MOGAD fulfill McDonald criteria for MS
ADEM-like lesions (bilateral, asymmetrical)	Yes (during relapse)	No	No	
Brainstem lesions	Yes	No	No	
Dawson’s fingers	No	Yes	Yes	
Subcortical U-fibre lesions	No	No	No	
≥ 1 lesion adjacent to lateral ventricle	Yes	Yes	Yes	
**MRI spine**				
Length of lesions	Short segment, multiple	Short segment, multiple	Longitudinally extensive, multiple	Longitudinally extensive central lesions with grey matter involvement favour MOGAD
Location of lesions	Cervical, thoracic	Cervical, thoracic	Cervical, thoracic	
Contrast enhancement	No	No	No	
**CSF**				
Pleocytosis	Nil	Not available	Lymphocytes 2X10^6^/L	In MOGAD, pleocytosis is variable & intrathecal OCB occur in 5–20% of patients. In MS, OCB present in 95%. Pleocytosis usually absent in MS
Protein	Normal	Not available	0.66 g/L (normal 0.15–0.45 g/L)	
Oligoclonal bands (OCB)	Negative	Not available	Positive

What is the likely explanation for the delayed diagnosis of MOGAD in these cases? Most likely these patients had MOGAD to begin with, but were misdiagnosed as MS. The ‘red flags’ in the history and investigations were probably not identified early (Table 1).

The large “fluffy” T2 lesions in MRI in case 1 [12, 13] are suggestive of MOGAD. In case 2, bilateral and recurrent ON should raise the possibility of MOGAD in the early stages, even though the initial MRI scan was not typical.

The development of novel autoimmune disorders following alemtuzumab therapy has been well reported. The most frequently reported condition is autoimmune thyroid disorders in approximately 40% of patients treated with alemtuzumab [14]. Other autoimmune disorders include immune thrombocytopenia and nephropathy [3]. The autoimmune diseases usually develop after a latent period ranging from months to years after treatment with alemtuzumab [3].

MOGAD has the potential to be misdiagnosed as MS due to certain overlapping clinical features. However, careful evaluation of clinical and radiological features should enable the clinician to distinguish between the two conditions. Several overlapping clinical features exist between the two conditions at onset, such as unilateral ON, however, onset with ON is more commonly seen in MOGAD than in MS (60–74% versus 15–20%) [8]. In MOGAD, optic neuritis is typically bilateral at onset and unilateral with relapses [7]. Short TM is reported to be an overlapping feature of MS and MOG antibody associated demyelination [15]. Moreover, both conditions can have a relapsing course, but MOGAD also has the potential to be monophasic, whilst MS also has the potential to be progressive [16]. In the Australian MOGAD cohort, short TM accounted for a higher proportion of relapses than longitudinally extensive transverse myelitis (LETM) [7]. In regard to radiological features at the disease onset, supratentorial radiological changes are present in a minority of MOGAD patients compared with MS, and those lesions tend to be atypical [8, 12]. In terms of supratentorial MRI lesions, Dawson’s fingers, as well as subcortical S-shaped and U-fibre lesions, are uncommon in MOGAD [16]. Spinal lesions in MOGAD typically extend over three or more vertebral segments [17]. However, a number of studies have shown that short TM is more common than initially thought in MOGAD, which can be a source of diagnostic confusion with MS [7, 15]. Recent studies have shown that 38% of MOGAD patients present with short TM and thoracic spinal cord is significantly more frequently involved in longitudinally extensive TM than short TM [10, 15].

Though it is well known that MOGAD is misdiagnosed as MS, testing all MS patients for MOG antibody is not practical, and likely of low yield given the rarity of MOGAD. Expert recommendations on the utility of MOG antibody testing have been published [18]. The key features that would warrant testing include clinical findings / clinical suspicion, neuroimaging not entirely typical of MS, and inadequate treatment response to usual MS disease modifying therapies [18]. The experts have also highlighted diagnostic “red flags” in relation to disease course, MRI, CSF and serology [18]. For example, borderline MOG-IgG and results from fixed cell-based assays should be treated with caution if the clinical picture is atypical.

Cerebrospinal fluid analysis plays a critical role in the diagnosis and several features help distinguish MOGAD from MS. Intrathecally restricted oligoclonal bands in CSF are uncommon (6–28%) in MOGAD [16, 17, 19], whereas in relapsing and remitting MS the test is positive in 95% of patients [16]. Intrathecal humoral response to neurotrophic viruses (measles, rubella, varicella zoster) is known as the MRZ reaction. A positive MRZ reaction is highly specific (97%) for MS whereas it is negative in > 98% MOGAD and neuromyelitis optica patients [20].

It is interesting to note that patient number 2 had bilateral ON and supratentorial brain lesions on the MRI at the onset of her illness. These features raise the possibility of MOGAD from the start. However, in case 1, the onset symptoms were limb weakness and ON and the MRI showed both supratentorial and infratentorial lesions. When MOGAD was diagnosed, she had ON and an increased number of lesions accompanied by oedema in the MRI scan. These features make one wonder whether she developed MOGAD later, but we cannot be certain with the available data.

Our literature search yielded a similar case report highlighting the lack of response to alemtuzumab in MOGAD [21]. This report describes a young woman who presented with myelitis followed by ON. She was diagnosed with MS and treated accordingly. Over the next nine years, she experienced 17 relapses. She was started on alemtuzumab therapy which paralleled with new relapses and multiple new brain and spinal lesions. At that stage, five serum samples obtained from the onset of the illness to the current stage were tested for MOG antibody, and all serum samples turned out to be positive [21]. This is a case of MOGAD misdiagnosed as MS, and it has some similarities to our cases. This case report along with our cases suggests that alemtuzumab is ineffective in MOGAD. Whether alemtuzumab therapy worsens MOGAD remains speculative at this stage but may warrant further research.

Patients with MOGAD treated with MS disease modifying therapies are known to relapse as was found in this case series and also reported previously [22]. Natalizumab is a monoclonal antibody against the α4β1 integrin and reduces the entry of CD4^+^ and CD8^+^ T lymphocytes into the CNS and has been shown to be quite effective in the treatment of relapsing remitting MS [23]. By virtue of its mechanism of action natalizumab can increase total peripheral lymphocyte counts, and it has also been shown to increase CD138^+^ plasma cells and immature CD19^+^CD10^+^ pre-B cells in the peripheral blood of natalizumab-treated patients [24]. Neuromyelitis optica spectrum disorder (NMOSD) and MOGAD are antibody mediated diseases and with increased CD138 + cells one could speculate increasing production of pathogenic antibodies, and therefore increased disease activity. In our series patients on natalizumab showed worsened disease activity and relapses. Others have shown similar responses to natalizumab in both NMOSD and MOGAD presentations where natalizumab was originally used for suspected MS [25]. It is evident from our reports above that if a patient with suspected MS on natalizumab shows increased disease activity, as well as considering anti natalizumab neutralizing antibodies and possibility of progressive multifocal leukoencephalopathy (PML), one should also have high vigilance in revisiting the original diagnosis and considering NMOSD or MOGAD as alternatives.

In regards to anti CD20 antibodies – they have been shown to be quite effective in MS [26] and AQP4-IgG-NMOSD [27, 28]. In MOGAD one large case series of 112 patients revealed that rituximab treatment led to a 37% decline in relapse rate, and after 2 years, 33% of patients were predicted to remain relapse-free [29] which is less effective than the effect of rituximab in MS and AQP4 positive NMOSD. Similarly, in an Australian study of 26 adults and 33 children with relapsing MOGAD, 1/7 patients did not respond to rituximab despite their B cells being depleted [7]. In line with this, Ocrelizumab – a humanised anti B cell depleting monoclonal antibody has been shown to be highly effective in MS [30], but it has not been studied in MOGAD. Despite the overlapping phenotypes between MS, AQP4 positive NMOSD and MOGAD, there appears to be differing pathophysiology in these diseases with different responses to B cell depletion – being highly efficacious in the former two, but not so in MOGAD.

Currently, MOGAD is treated with intravenous or oral pulsed corticosteroids with a cautious steroid taper. While half the patients may be monophasic, the rest may relapse and require maintenance immunosuppression. There is recent data that maintenance IVIG is efficacious in preventing future attacks [22]. Other agents that also reduce annualized relapse rate include azathioprine, rituximab and mycophenolate mofetil [22]. In a case series, Elsbernd and colleagues reported that tocilizumab (an IL-6 inhibitor) may be a promising treatment option for patients with relapsing MOGD who have not responded to other therapies [31]. Tocilizumab was not considered in the 3 cases here due to its lack of availability at the time of the case presentations.

In conclusion, our cases demonstrate that alemtuzumab is ineffective in the management of MOGAD, but steroids, plasma exchange and IVIG are helpful. We also suggest that those patients diagnosed with MS but with atypical clinical features and demonstrating a lack of response to alemtuzumab should be tested for MOG antibody. These cases also highlight the importance of clinical suspicion to enable early diagnosis, particularly when patients present with atypical features for MS, such as bilateral ON and long spinal lesions.

## Data Availability

Not applicable.

## Abbreviations

ADEM: Acute demyelinating encephalomyelitis
EDSS: Expanded disability status scale
IVIG: Intravenous immunoglobulin
MOG: Myelin Oligodendrocyte Glycoprotein
MOGAD: Myelin Oligodendrocyte Glycoprotein Antibody Associated Disease
MRI: Magnetic resonance imaging
ON: Optic neuritis
RRMS: Relapsing–remitting multiple Sclerosis
TM: Transverse myelitis
